# Characterization of Host and Bacterial Contributions to Lung Barrier Dysfunction Following Co-infection with 2009 Pandemic Influenza and Methicillin Resistant *Staphylococcus aureus*

**DOI:** 10.3390/v11020116

**Published:** 2019-01-29

**Authors:** Michaela E. Nickol, Justine Ciric, Shane D. Falcinelli, Daniel S. Chertow, Jason Kindrachuk

**Affiliations:** 1 Laboratory of Emerging and Re-Emerging Viruses, Department of Medical Microbiology, University of Manitoba, Winnipeg, MB R3E 0J9, Canada; nickolm@myumanitoba.ca (M.E.N.); ciricj@myumanitoba.ca (J.C.); 2 Department of Microbiology and Immunology, University of North Carolina Chapel Hill School of Medicine, Chapel Hill, NC 27599, USA; shane_falcinelli@med.unc.edu; 3 Laboratory of Immunoregulation, National Institute of Allergy and Infectious Diseases, National Institutes of Health, Bethesda, MD 20892, USA; chertowd@cc.nih.gov; 4 Critical Care Medicine Department, National Institutes of Health Clinical Center, Bethesda, MD 20892, USA

**Keywords:** influenza, *Staphylococcus aureus*, co-infection, 2009 pandemic, alveolar epithelial cells, kinome, virulence factors, barrier function

## Abstract

Influenza viruses are a threat to global public health resulting in ~500,000 deaths each year. Despite an intensive vaccination program, influenza infections remain a recurrent, yet unsolved public health problem. Secondary bacterial infections frequently complicate influenza infections during seasonal outbreaks and pandemics, resulting in increased morbidity and mortality. *Staphylococcus aureus*, including methicillin-resistant *S. aureus* (MRSA), is frequently associated with these co-infections, including the 2009 influenza pandemic. Damage to alveolar epithelium is a major contributor to severe influenza-bacterial co-infections and can result in gas exchange abnormalities, fluid leakage, and respiratory insufficiency. These deleterious manifestations likely involve both pathogen- and host-mediated mechanisms. However, there is a paucity of information regarding the mechanisms (pathogen- and/or host-mediated) underlying influenza-bacterial co-infection pathogenesis. To address this, we characterized the contributions of viral-, bacterial-, and host-mediated factors to the altered structure and function of alveolar epithelial cells during co-infection with a focus on the 2009 pandemic influenza (pdm2009) and MRSA. Here, we characterized pdm2009 and MRSA replication kinetics, temporal host kinome responses, modulation of MRSA virulence factors, and disruption of alveolar barrier integrity in response to pdm2009-MRSA co-infection. Our results suggest that alveolar barrier disruption during co-infection is mediated primarily through host response dysregulation, resulting in loss of alveolar barrier integrity.

## 1. Introduction

Influenza A viruses (IAV) have posed a persistent threat to global public health for centuries, through both recurrent seasonal epidemics and sporadic pandemic outbreaks [1]. Approximately 10% of the global population is infected with an influenza virus annually, resulting in an estimated 3–5 million severe infections and 300,000–500,000 deaths [1,2,3]. Initial signs and symptoms include acute onset of high fever, headache, cough, myalgias, and fatigue [4,5]. IAV is a self-limiting infection in most healthy adults, predominantly affecting the upper respiratory tract, and typically resolves within seven days of symptom onset [4,5]. However, severe infections progress to the lower respiratory tract, resulting in increased risk of respiratory failure and death. Populations at increased risk of severe influenza infection include infants, the elderly, pregnant women, and individuals with pre-existing respiratory, cardiac, neurological, or immunosuppressive conditions [5,6].

There is an increasing appreciation that a large percentage of severe or fatal influenza infections is associated with secondary bacterial infections [7]. The contribution of bacterial infection to influenza morbidity and mortality was well documented throughout the 1918 “Spanish” influenza pandemic and in all subsequent influenza pandemics over the past century [8]. Modern analyses of lung tissue and review of historical autopsy data from fatal 1918 influenza infections demonstrated that 95% of lethal cases were complicated by bacterial co-infection, primarily due to *Streptococcus pneumoniae* and *Staphylococcus aureus* [9,10,11]. During the 1957 and 1968 influenza pandemics, secondary bacterial pneumonia also caused significant morbidity and mortality, with *S. aureus* and *S. pneumoniae* being the predominant bacterial pathogens [9,12,13,14,15,16]. During the 2009 influenza pandemic, up to 34% of severe influenza infections managed in intensive care units and up to 55% of fatal cases were complicated by bacterial co-infections [17,18,19,20]. It is estimated that approximately 65,000 influenza- and pneumonia-related deaths occur in the U.S. each year [17]. *S. aureus*, including methicillin-resistant *S. aureus* (MRSA), is highly prevalent in severe IAV-bacterial co-infection in adults and infants [21,22,23,24].

Host and pathogen molecular mechanisms that contribute to severe influenza-bacterial infections in the lower respiratory tract are poorly understood. Excessive mucus production and impaired mucociliary clearance in response to IAV infection facilitates bacterial colonization of the lower respiratory tract, and respiratory epithelial cell barrier breakdown predisposes to bacterial invasion [7,17,21,25]. Influenza infection may also enhance bacterial adhesion to cells through the incorporation of hemagglutinin into the host cell membrane, promoting bacterial cell attachment [25,26]. These events, in conjunction with respiratory epithelial cell barrier breakdown, are likely critical to the development of secondary bacterial infections [25]. Type I and type II alveolar epithelial cells, responsible for physiology gas-exchange and surfactant production, respectively, become infected by influenza viruses, and altered alveolar-capillary membrane function results in impaired oxygen exchange and lung injury [27,28]. However, molecular mechanisms contributing to (1) bacterial replication, (2) bacterial virulence factor expression, and (3) host cell signaling in the context of IAV co-infection to epithelial cell barrier breakdown have not been fully elucidated [25]. Understanding the contribution of these factors to co-infection pathogenesis may yield novel therapeutic targets for treatment of IAV and bacterial co-infection.

As the pathophysiology of severe influenza-bacterial co-infections is primarily associated with the lower respiratory tract, we sought to characterize the contributions of viral-, bacterial-, and host-mediated factors to alveolar cell dysfunction. For this analysis, we employed human adenocarcinoma A549 alveolar epithelial cells to characterize host- and pathogen contributions directly in a relevant and well-characterized alveolar epithelial cell line. Further, A549 cells have been used extensively for the analysis of host responses to influenza virus infection [29,30,31,32,33]. We studied (1) the impact of IAV-infection on MRSA replication kinetics in A549 cell culture, (2) the host cell response to IAV, MRSA, or co-infection by analyzing temporal intracellular kinome responses, (3) the modulation of MRSA virulence factors related to adhesion and invasion in the presence or absence of IAV co-infection by RT-qPCR, and (4) alveolar epithelial barrier function and integrity during IAV, MRSA, or co-infection using electric cell-substrate impedance sensing (ECIS).

## 2. Materials and Methods

### 2.1. Virus, Bacteria, and Cell Conditions

The 2009 pandemic H1N1 Influenza A/Mexico/4108/09 (pdm2009) was kindly provided by Dr. Kevin Coombs (University of Manitoba, Canada). Virus stocks were grown in Madin–Darby canine kidney cells and concentrated following ultracentrifugation on a 35% sucrose cushion, kept at −80 °C. Viral titers were determined via plaque assay [34]. MRSA USA300 (herein referred to as MRSA) was kindly provided by Dr. George Zhanel (University of Manitoba, Canada). MRSA inocula were generated following growth to mid-log phase in tryptic soy broth (TSB; Hardy Diagnostics, Santa Maria, CA, USA). Human A549 adenocarcinomic alveolar basal epithelial cells were grown in DMEM (Gibco, Grand Island, NY, USA) supplemented with 10% fetal bovine serum (Gibco, Grand Island, NY, USA) and 1% penicillin-streptomycin (HyClone Laboratories, South Logan, UT, USA) at 37 °C and 5% CO_2_. Normal human bronchial epithelial cells infected with human telomerase and CDK4-epxressing retrovirus (HBEC-3KT) were kindly provided by Dr. Neeloffer Mookherjee (University of Manitoba, Canada). Cells were grown in Airway Epithelial Basal Cell Medium fully supplemented with the Bronchial Epithelial Cells Growth Kit (ATCC).

### 2.2. Viral and Bacterial Infection of Alveolar Epithelial Cells

For infectious assays, alveolar epithelial cells were seeded at ~95% confluence in DMEM supplemented with 2% FBS 1 day prior to infection. Cells were infected with pdm2009 at a multiplicity of infection (MOI) of 0.01 or mock-infected with DMEM supplemented with 2% FBS for 1 h with gentle rocking every 15 min. Following infection, viral inocula were aspirated from cells and replenished with fresh DMEM supplemented with 2% FBS. Cells were rested for 24 h post-pdm2009 infection. Cells were then infected with mid-log phase MRSA USA300 or mock-infected 24 h post-pdm2009 addition (DMEM supplemented with 2% FBS) for 1 h with gentle rocking every 15 min to ensure equal distribution of bacteria. Bacterial MOIs of 0.1 and 0.01 were used in this investigation and were achieved by serial dilution of mid-log phase culture in DMEM supplemented with 2% FBS. Bacterial inocula were aspirated from cells and replaced with fresh DMEM supplemented with 2% FBS. Cells were harvested at 0, 1, 4, 8, 12, 16, 20, and 24 h post-MRSA infection for further investigation of bacterial replication kinetics, bacterial virulence factors’ expression, and kinome analysis. Infections of HBEC-3KT cells (kindly provided by the Mookherjee laboratory) utilized the same conditions with the exception that cells were maintained in Airway Epithelial Basal Cell Medium supplemented with 6 mM l-glutamine.

### 2.3. Quantification of Bacterial and Viral Replication Kinetics

Enumeration of the total number of adherent and internalized bacteria was determined at 0, 1, 4, 8, 12, 16, 20, and 24 h post-MRSA infection. Media was aspirated from wells, and cells were washed at least 2× with PBS prior to harvest for bacterial enumeration. Alveolar epithelial cells were lysed with 0.025% TritonX-100 (VWR Life Science, Solon, OH, USA). Cell lysates (incl. intact bacteria) were collected, and MRSA colony forming units were enumerated by standard bacterial plating on tryptic soy agar (TSA; MP Biomedicals, LLC, Solon, OH, USA). Viral replication was quantified by RT-qPCR) in supernatant samples from pdm2009-MRSA-infected A549 cells. Total RNA was extracted from the supernatants with the PureLink Viral RNA/DNA Mini Kit (Life Technologies, Burlington, ON, USA) according to the manufacturer’s instructions. Reverse transcription of total RNA was performed with primers specific for the pdm2009 H1N1 HA sequence using the Superscript IV first-strand cDNA synthesis kit (Life Technologies, Burlington, ON, USA). RT-qPCR was performed on an Applied Biosystems QuantStudio 6 Flex Real-Time PCR System, and each sample was run in duplicate with the PowerUp SYBR Green PCR master mix (Life Technologies, Burlington, ON, USA). Quantification of viral copy number was accomplished by comparison of RT-qPCR results to an established external standard of viral copy number.

### 2.4. Kinome Peptide Array Analysis

Kinome peptide array analysis was performed as previously described [35,36]. Briefly, IAV-, MRSA-, IAV-MRSA-, and mock-infected alveolar epithelial cells were scraped and pelleted by gentle centrifugation at 4, 8, 12, 16, 20, and 24 h post-MRSA addition. Cell pellets were treated with kinome lysis buffer (20 mM Tris-HCl, pH 7.5, 150 mM NaCl, 1 mM EDTA, 1 mM EGTA, 1% Triton X-100, 2.5 mM sodium pyrophosphate, 1 mM Na3VO4, 1 mM NaF, 1 μg/mL leupeptin, 1 μg/mL aprotinin, 1 mM phenylmethylsulfonyl fluoride) and incubated on ice for 10 min. Cell lysates were clarified by centrifugation at 14,000 rpm. Cell lysates were transferred to fresh microcentrifuge tubes, and the total protein concentrations were measured using the Pierce BCA Protein Assay Kit. Activation mix (50% glycerol, 50 μM ATP, 60 mM MgCl_2_, 0.05% Brij 35, 0.25 mg/mL bovine serum albumin) was added to the equivalent amounts of total protein (100 µg) for each sample, and total sample volumes were matched by the addition of kinome lysis buffer. Samples were spotted onto kinome peptide arrays (JPT Peptide Technologies GmbH, Berlin, Germany) and incubated for 2 h at 37 °C and 5% CO_2_. Following incubation, arrays were washed once with PBS containing 1% Triton X-100, followed by a single wash in deionized H_2_O. Arrays were stained with PRO-Q Diamond phosphoprotein stain (Invitrogen, Carlsbad, CA, USA) for 1 h with gentle agitation. Arrays were subsequently destained (20% acetonitrile, 50 mM sodium acetate, pH 4.0) 3 times × 10 min each with the addition of fresh destain each time. A final 10 min wash was performed with deionized H_2_O. Arrays were dried by gentle centrifugation. Array images were acquired using a PowerScanner microarray scanner (Tecan, Morrisville, NC, USA) with a 580-nm filter to detect dye fluorescence. Signal intensity values were collected using Array-Pro Analyzer version 6.3 software (Media Cybernetics, Rockville, MD, USA). Kinome data analysis was performed using the Platform for Integrated, Intelligent Kinome Analysis 2 (PIIKA2) software (available online: http://saphire.usask.ca/saphire/piika), as described previously [37]. Additional heatmaps were derived using the Heatmapper software suite [38]. Phosphorylation fold changes were validated using the Proteome Profiler Human Phospho-Kinase Array Kit according to the manufacturer’s instructions (R&D Systems, Minneapolis, MN, USA).

### 2.5. Pathway Overrepresentation and Gene Ontology Analysis

Pathway overrepresentation and gene ontology analyses of differentially-phosphorylated proteins were performed using InnateDB software as described previously [35,36,39]. Input data were limited to peptides that demonstrated statistically-significant changes in expression as compared to their respective time-matched mock-infected controls, as described previously [40]. Protein identifiers, phosphorylation fold change values (>1), and *p*-values (<0.05) were uploaded to InnateDB.

### 2.6. RNA Extraction, cDNA Synthesis, and Quantitative qPCR

At each time point, samples were collected to determine the modification of bacterial virulence factors. Three biological replicates were collected per sample. Media was aspirated from the wells, and the cell monolayers were gently scraped, then pelleted via centrifugation for 10 min at 1200 rpm. The cell pellets were stored at −80 °C until RNA extraction. Bacterial RNA extraction was performed via standard TRIzol-chloroform extraction (Ambion, Carlsbad, CA, USA). Equivalent amounts of RNA from each biological replicate were used for cDNA synthesis using the QuantiNova reverse transcription kit (Qiagen, Hilden, Germany) with random primers. RT-qPCR was performed on the Applied Biosystems QuantStudio 6 Flex Real-Time PCR system (Life Technologies, Burlington, Ontario) using PowerUp SYBR Green Master Mix (Applied Biosystems, Austin, TX, USA) as a detection method. Each biological replicate was run in at least technical duplicate, and non-template controls were included during each run. Melt curve analysis was performed to ensure amplification specificity. Bacterial gene expression was quantified through comparison to the MRSA housekeeping gene 16S [41], and relative fold change in expression was calculated using the 2^−ΔΔ*C*T^ method. Relative fold change values represent pdm2009-MRSA (normalized to 16S)/MRSA-alone (normalized to 16S). Primer sequences are presented in Table S1.

### 2.7. Barrier Integrity Determination

The ECIS Zθ, 96W Array Station and 96W20idf PET plates (Applied BioPhysics, Troy, NY, USA) were employed to quantify changes in the barrier integrity of alveolar epithelial cells during pdm2009-MRSA co-infection. A549 cells were seeded in 96W20idf PET plates at a concentration of 50,000 cells/mL and were rested in the 96W Array Station (Applied BioPhysics, Troy, NY, USA) for 24 h at 37 °C with 5% CO_2_ prior to pdm2009 infection. Cells were subsequently infected with pdm2009 (MOI of 3.0 or 0.1) or mock-infected (DMEM supplemented with 2% FBS) followed by resting for 24 h. Viral- and mock-infected cells were subsequently infected with mid-log phase MRSA USA300 (MOI of 0.1 or 0.01) or mock-infected (DMEM supplemented with 2% FBS) 24 h post-pdm2009 infection. Resistance measurements were acquired during the duration of the entire experiment (72 h). Control conditions included: (i) cells infected with pdm2009-alone (MOI of 3.0, 0.1, or 0.01); (ii) cells infected with MRSA-alone (MOI of 0.01); (iii) mock-infected cells (background barrier resistance); and (iv) cells treated with 1% Triton-X100 (positive control for barrier dysfunction).

## 3. Results

### 3.1. MRSA Replication Kinetics Are Similar during Bacterial Infection-Alone and pdm2009-MRSA Infection

**Figure 1:**
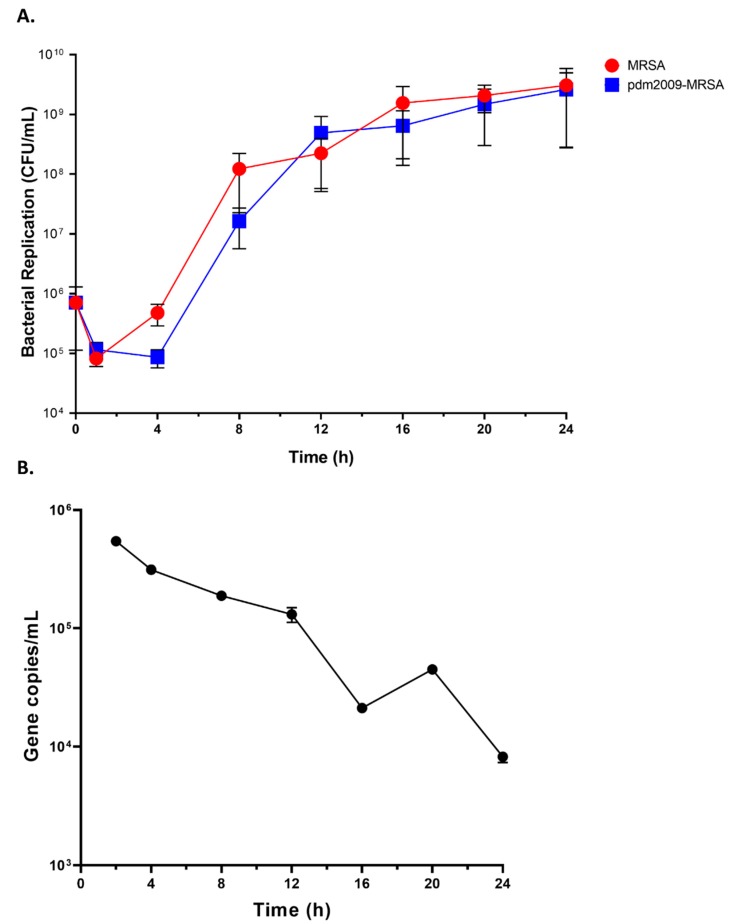
Pathogen replication kinetics during bacterial infection and influenza-bacterial co-infection. A549 cells were infected with 2009 pandemic influenza (pdm2009) (MOI 0.1) or mock-infected, followed by MRSA infection 24 h later (MOI 0.1). (**A**) Alveolar epithelial cells were selectively lysed at the indicated time points, and CFU were quantified by standard bacterial plating. (**B**) Supernatants were harvested for isolation of viral RNA prior to quantification by RT-qPCR. Error bars represent SEM calculated from at least three biological replicates. Error bars for some of the time points are not visible due to the y-axis scale. No significant differences were found between groups as assessed by two-way ANOVA and significance testing by Tukey’s test.

We first sought to characterize bacterial replication kinetics in human lung epithelial cells during IAV co-infection. Although IAV-bacterial co-infections can result in increased lung pathology in humans and nonhuman primates [42,43,44], there is little information available regarding the relation of bacterial replication kinetics to increased disease severity. To address this, we temporally-enumerated MRSA replication in alveolar epithelial cells during MRSA and pdm2009-MRSA infections. MRSA was added to mock-infected or pdm2009-infected cells 18 h post-infection. Timing was based on observational data from human patients with influenza-bacterial co-infections during the 2009 H1N1 pandemic where bacterial co-infection commonly occurred during the peak of viral infection [17]. The total number of adherent and internalized bacteria in alveolar epithelial cells was enumerated through standard bacterial plating. Although there was a trend towards faster bacterial replication in MRSA-alone infection as compared to pdm2009-MRSA infection at 4 h and 8 h post-MRSA infection, there were no statistically-significant differences in MRSA replication between the two conditions at any time point (Figure 1). Cells infected with MRSA-alone entered the exponential growth phase at 1 h post-infection and the stationary phase at 16 h post-infection. This was largely mirrored in the pdm2009-MRSA-infected cells. Bacterial colony counts began increasing exponentially at 4 h post-infection and entered the stationary phase beginning at 16 h post-infection. A similar pattern of MRSA replication in the presence or absence of pdm2009 co-infection was also found in HBEC-3KT cells (Figure S1). This suggests that bacterial replication kinetics are not impacted during influenza co-infection in anatomically- and physiologically-distinct regions of the lungs. To confirm that A549 cells were productively infected by pdm2009, supernatants were harvested from the pdm2009-MRSA-infected cells at the same time points as those used for CFU determination. Influenza virus gradually decreased throughout the course of co-infection (Figure 1B).

### 3.2. Temporal Analysis of Host Kinome Responses During pdm2009-MRSA Infection

**Figure 2:**
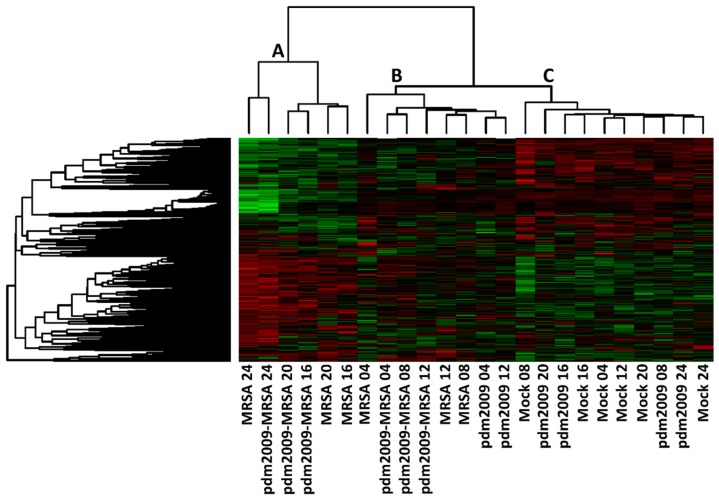
Hierarchical clustering of temporal kinome responses of pdm2009, MRSA, and pdm2009 MRSA-infections in alveolar epithelial cells. Cells were plated 24 h prior to initial infection with pdm2009. Cells were infected or mock-infected with pdm2009 (MOI 0.1). Twenty four h post-pdm2009 infection, MRSA was added to cells at an MOI of 0.1. Cells were harvested for kinome analysis at the indicated time points. MRSA-alone-, pdm2009-alone-, and mock-infected time-matched samples were also collected at the indicated time points. **A**–**C** designate the three major dataset clusters as identified following hierarchical clustering.

As bacterial replication rates were virtually identical between the MRSA-alone infection and pdm2009-MRSA infection, we next sought to address whether aberrant cell-mediated immune responses may contribute to IAV and bacterial co-infection pathogenesis. We performed temporal kinome analysis of pdm2009-, MRSA-, and pdm2009-MRSA-infected alveolar epithelial cells. We postulated that the activation state of host cell signaling responses or individual cellular kinases could provide insight into differential cellular responses found within co-infected cells as compared to pdm2009- or MRSA-alone. Time-matched mock-infected control cells served as controls. Naive A549 cells were initially infected with pdm2009 (MOI 0.1) or mock-infected and rested for 24 h prior to bacterial infection. MRSA addition to MRSA-infected and pdm2009-MRSA-co-infected cells was designated as Time 0. Cells were harvested at various post-MRSA infection time points ranging from 4 h post-MRSA addition to 24 h post-infection. Both pdm2009-alone infected cells and mock-infected control cells were treated with MRSA-free infection inoculum at Time 0 to normalize cellular responses that may have been induced through the physical stress of the inoculum addition. Time-matched pdm2009-, MRSA-, and mock-infected control cells were collected throughout the duration of the experiment. Cell lysates were subsequently probed to quantitate host kinome responses by kinome peptide arrays. This analysis relies on the phosphorylation of specific kinase targets (immobilized peptides) on the arrays by active kinases in a cell lysate [45,46]. Data from our arrays, comprised of 309 unique kinase recognition sequences related to a broad spectrum of cell signaling pathways and processes, was analyzed using the Platform for Intelligent, Integrated Kinome Analysis 2 (PIIKA 2) software tool [37]. Hierarchical clustering analysis of the kinome data is presented in Figure 2. Overall, the kinome datasets clustered into three major clusters, which were primarily grouped based on post-MRSA infection time points. From left to right, the first cluster consisted of the 16, 20, and 24 h post-infection MRSA-alone and pdm2009-MRSA co-infection datasets (denoted as A). The second major cluster was comprised of the 4 h and 12 h MRSA-, pdm2009-, and pdm2009-MRSA-infected samples, as well as the 8-h MRSA- and pdm2009-MRSA-infected samples (denoted as B). In the third cluster, all of the mock-infected datasets clustered together along with the 8, 16, 20, and 24 h post-infection pdm2009-alone infected datasets (denoted as C). Clusters B and C were more similar to each other than to the samples from Cluster A. These data suggested that the host kinome responses of the MRSA- and pdm2009-MRSA-infected samples from 16 h onwards were highly conserved between the two conditions and differentiated strongly from all other infection conditions and post-infection time points.

**Figure 3:**
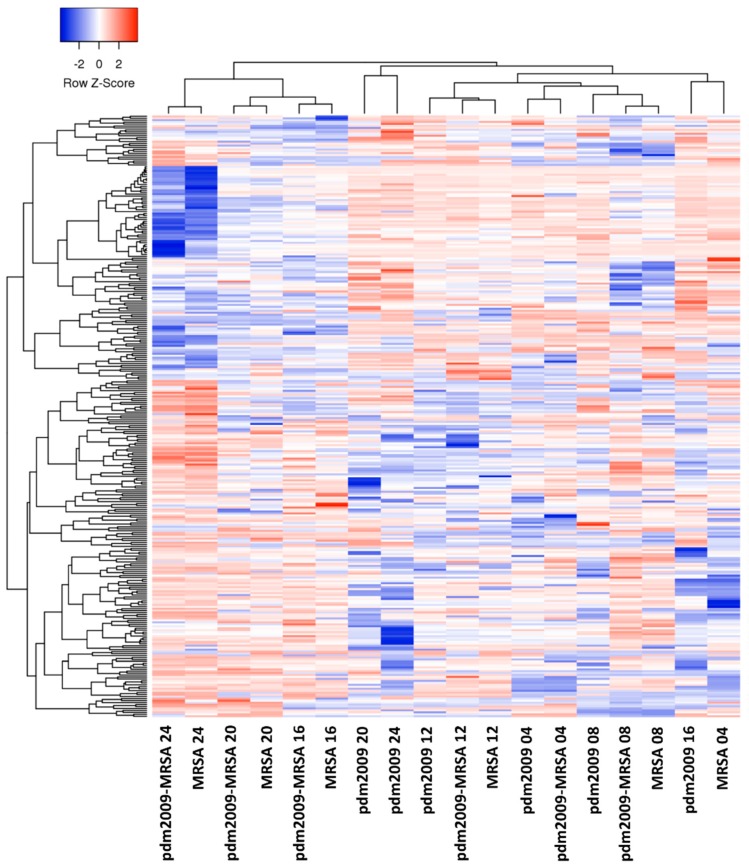
Background-subtracted temporal kinome responses of pdm2009, MRSA, and pdm2009-MRSA infection. Mock-infected kinome responses were subtracted from the time-matched infected samples. Fold change phosphorylation values are plotted for all kinase recognition sequences on the kinome peptide arrays. Clustering analysis was performed with the Heatmapper software suite. Z-score values represent fold change differences in phosphorylation as compared to the time-matched mock-infected control cells.

To gain further insight into the similarities and/or differences in host kinome responses during pdm2009-MRSA co-infection as compared to infection by either pathogen alone, we performed biological subtraction of the time-matched mock-infected kinome datasets from their respective infected counterparts. Respective hierarchical clustering analysis of phosphorylation fold changes following mock-infected background subtraction is presented in Figure 3. Notably, the 16–24 h MRSA- and pdm2009-MRSA-infected datasets grouped together independent of the time-matched pdm2009-infected datasets. In contrast, at 4 h post-MRSA infection, the pdm2009-MRSA and pdm2009-alone datasets clustered together independent of the time-matched MRSA-alone dataset. However, from 8–12 h post-MRSA infection, the MRSA-alone, pdm2009-alone, and pdm2009-MRSA datasets clustered together. These data suggested that a transition phase occurred in the host cellular response from 8–12 h post-MRSA infection, whereby the host cellular response switches from an IAV-dominated to a MRSA-dominated response. To provide additional validation of the kinome data, we performed phospho-Western blot analysis with Phospho-kinase Proteome Profiler Arrays (R&D Systems). Phosphorylations events that were conserved between the upregulated phosphorylation on the arrays (fold change >1.5; *p*-value < 0.05) and the phospho-Western blots are presented in Table 1.

We next sought to identify host cell signaling responses or biological networks in the pdm2009-MRSA-infected alveolar cells that were selectively modulated during the 8–12 h post-MRSA infection transition phase (Figure 3). Pathway over-representation analysis at 8-h post-MRSA infection resulted in the identification of numerous pathways that were selectively upregulated as compared to the mock-infected cells (Table S2). Multiple apoptosis-related pathways were identified to be activated at this time point including p75(NTR)-, NRAGE-, NRIF-, NADE-, Tsp-1- and BH3-mediated signaling events. In addition, signaling pathways directly related to cell-cell contacts were also identified including alpha 6-beta 4 integrin-, and ephrin-mediated signaling events. Viral infection-related signaling pathways were also identified (incl. type I interferon (IFN) and inflammatory response-related signaling events). At the 12-h post-MRSA infection time point, the host response was dominated by NOTCH-related signaling and antiviral response-mediated events (incl. TRAF6-mediated IRF7 activation and IFN-related responses). As predicted from our hierarchical clustering analyses, there were strong similarities in the overrepresented signaling pathways found between the pdm2009-MRSA co-infection and MRSA-alone infection samples from 16–24 h post-MRSA addition. Upregulated pathways were primarily related to pro-apoptotic (e.g., p53- and caspase-mediated responses), cytokine signaling (e.g., TNFα; IL1; NFκB), innate immune response (e.g., TLR signaling; IFNβ), and wound healing (e.g., TGFβ-mediated signaling). In contrast, the pdm2009-alone infection samples were dominated by IFN-, JAK/STAT-, and IL4-mediated signaling during this time frame with p53-mediated signaling only being overrepresented at the 24-h time point. To provide additional clarity regarding potential differences in host responses between MRSA-alone and pdm2009-MRSA infection during the 8–12-h transition period, we directly compared the pdm2009-MRSA kinome responses to MRSA-alone responses (Table S3). At the 8-h time point, apoptosis-related signaling pathways were over-represented in pdm2009-MRSA-infected cells as compared to cells infected with MRSA-alone, suggesting that co-infection may result in an earlier activation of apoptosis in alveolar epithelial cells as compared to MRSA. At 12 h post-infection, there were fewer total differentially-upregulated pathways between the two infection conditions. Pathways related to TRAF6-, p75NTR, and apoptosis were differentially upregulated during pdm2009-MRSA infection; however, there was no clear over-representation of a particular biological response (e.g., apoptosis).

In addition, we also performed gene ontology (GO) analysis to identify biological processes that were overrepresented within our pdm2009-MRSA kinome data during the 8–12-h transition period (Table S4). While the majority of the biological processes identified mirrored those found in the pathway analysis (i.e., apoptosis- and IFN-related cellular responses) or were directly related to cellular damage responses (e.g., ATP catabolism, unfolded protein response, DNA damage), IL6- and IL8-related cellular responses were also identified, suggesting a potential role of these immune mediators in the host response to IAV bacterial co-infections. The greatest number of significantly-modulated signaling pathways was identified at 24 h post-MRSA infection. Many of the identified pathways were related to pro-inflammatory responses (incl. TNF- and IL1-mediated signaling), general innate immune responses (incl. NFκB-, TLR-, and RIG-1/MDA5-mediated signaling), apoptosis, wound healing (e.g., TGFβ signaling), and cell–cell contacts (e.g., alpha 6-beta 4 signaling). Collectively, our host kinome response data demonstrated that pdm2009-MRSA infection resulted in the selective activation of host cell signaling events largely related to apoptosis, cell–cell contacts and innate immune responses. While the overrepresented cell signaling pathways in the pdm2009-MRSA-infected samples resembled those found in both the pdm2009- and MRSA-alone infections during the 8–12-h time period, the cell signaling responses in the co-infections strongly resembled those of the MRSA-alone infections from 16 h onwards.

### 3.3. Bacterial Invasion- and Attachment-Related Virulence Factor Expression Patterns Are Modulated Early during pdm2009-MRSA Infection

**Figure 4:**
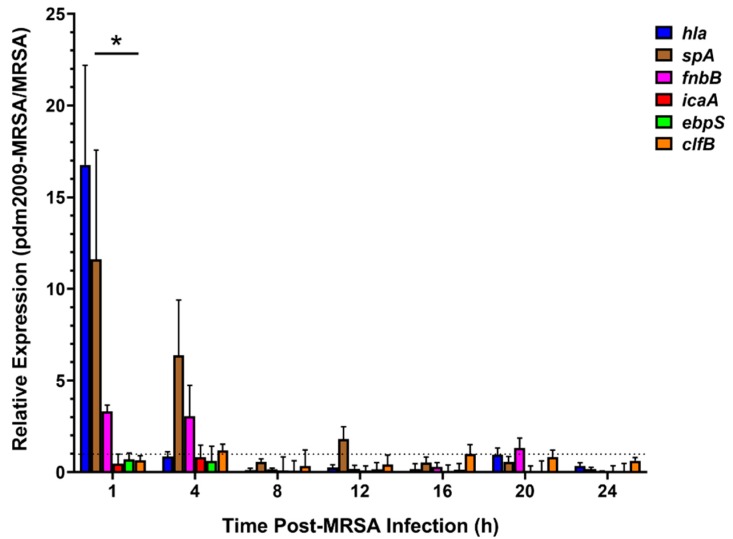
pdm2009-MRSA co-infection alters bacterial virulence factor expression in alveolar epithelial cells as compared to MRSA infection alone. MRSA virulence factor expression fold change values for co-infected samples relative to MRSA-alone-infected cells. Error bars represent SEM calculated from at least three biological replicates. Relative expression fold changes represent pdm2009-MRSA vs. MRSA infection alone and were calculated by the 2^−ΔΔCT^ method. Comparison between groups was assessed by two-way ANOVA and significance testing by Tukey’s test, *p < 0.05.

As our prior analysis had focused on the potential role of selective host response modulation in IAV bacterial co-infection pathogenesis, we next sought to characterize the potential role of selective modulation of bacterial virulence factors to pathogenesis. We employed RT-qPCR to examine differential modulation of MRSA virulence factor gene expression in the presence or absence of pre-existing influenza virus infection in alveolar epithelial cells. We focused on MRSA virulence factor genes related to host cell adhesion and invasion for our analysis. Overall, the expression of virulence factors in the pdm2009-MRSA-infected cells was largely repressed (<1) relative to MRSA-alone from 8 h onwards (Figure 4). In contrast, the expression of *hla*, *spa*, and *fnbB* was highly upregulated in the co-infected A549 cells relative to MRSA-alone at 1 h post-MRSA addition. The expression of *spA* and *fnbB* remained upregulated in the co-infected cells at 4 h post-infection, while the expression of *hla* was similar to MRSA-alone. This pattern of differential gene expression coincided with the early exponential phase of MRSA replication in our infected cells (Figure 1). Taken together, our data suggest that MRSA virulence factors may contribute to pdm2009-MRSA co-infection pathogenesis during the early phase of bacterial attachment and entry in alveolar epithelial cells.

### 3.4. MRSA-Alone and pdm2009-MRSA Infection Result in Alveolar Epithelial Cell Barrier Dysfunction

Our host kinome data suggested that pdm2009-MRSA co-infection-mediated modulation of host cell signaling responses shifted to a MRSA-dominated response during the 8–12-h post-MRSA addition period. Thus, we next examined whether the MRSA-dominated host response observed in the kinome data also dominated barrier function disruption. To assess this, we incorporated ECIS to characterize alveolar epithelial cell barrier function in response to pdm2009-MRSA infection by measuring temporal changes in resistance [47].

**Figure 5:**
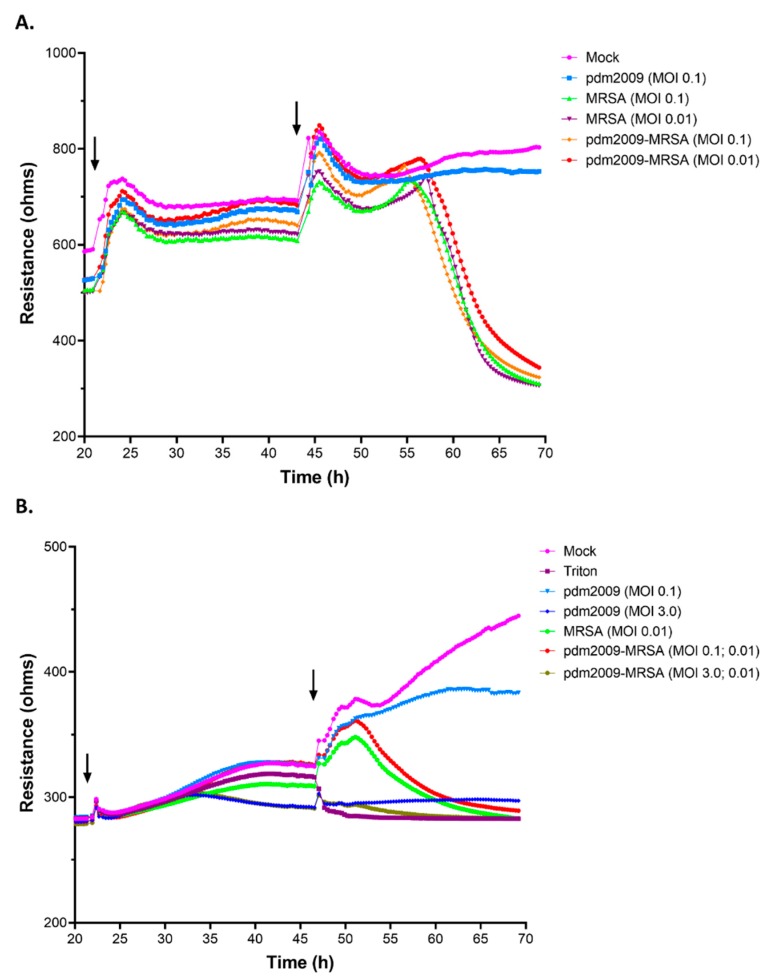
pdm2009-MRSA infection decreases barrier function in alveolar epithelial cells. Median resistance values have been plotted for all data points obtained during the experiment. Error bars have been removed to allow for clear visualization of all datasets, but were consistent across all biological replicates. A549 cells were plated 24 h prior to initial infection with pdm2009. Cells were infected or mock-infected with pdm2009, and MRSA was added to cells 24 h later. MRSA-alone, pdm2009-alone, 1% triton, and mock-infected time-matched conditions were also analyzed at the indicated time points. MOI values in parentheses signify the MRSA MOI utilized for infection. (**A**) Resistance data following infection with pdm2009 (MOI 0.1) and/or MRSA (MOI 0.1 or 0.01). (**B**) Resistance data following analysis with high MOI pdm2009 (MOI 0.1 or 3) and/or low MOI MRSA (MOI 0.01). Resistance data represent the median of at least three biological replicates with at least six technical replicates per sample per biological replicate. Arrows designate the addition of pdm2009 (first arrow) and MRSA or triton (second arrow) to the cells.

Alveolar epithelial cells were initially plated and rested overnight prior to pdm2009 infection (0 h). Following the overnight rest, cells were infected with pdm2009 (first arrow; Figure 5). Mock-infected control cells were treated with media alone. IAV- and mock-infected cells were subsequently infected with mid-log phase MRSA or mock-infected with media alone (second arrow; Figure 5), and resistance was continually monitored for the duration of the experiment. A summary of the data is presented in Figure 5, beginning just prior to the initial infection with pdm2009 (20 h). Mock-infected cells were left untreated throughout the duration of the experiment (negative control).

Infection of alveolar epithelial cells with pdm2009-alone at a MOI of 0.1 resulted in no changes to resistance during the first 24 h of infection. Only a small loss in resistance was found following the addition of mock-infected inoculum at 43 h (Figure 5A). The addition of MRSA (MOI 0.1) to mock-infected alveolar epithelial cells at 43 h resulted in the loss of resistance of the alveolar epithelial monolayer beginning at 57 h. Infection with pdm2009-MRSA (MOI 0.1) resulted in a nearly identical loss in resistance across the alveolar epithelial cells as that for infection with MRSA-alone. We also examined how a lower MOI of MRSA would affect the trends in resistance across our alveolar epithelial cells. Losses in resistance across the alveolar epithelial monolayers were similar between the two MOIs of MRSA tested (0.1 and 0.01). In contrast, the trend in resistance measurements differed slightly between the two MOIs of MRSA during co-infection. As these results suggested that pdm2009-MRSA co-infection-mediated disruption of alveolar epithelial barrier function largely resembled that of MRSA infection alone, we sought to expand on these observations. We examined pdm2009-MRSA co-infection using higher MOI pdm2009 and/or lower MOI MRSA inocula (Figure 5B). Infection with pdm2009-MRSA (MOI 0.1; MOI 0.01) resulted in a nearly identical loss in resistance across the alveolar epithelial cells as that for infection with MRSA-alone. We also examined how a higher MOI of pdm2009 would affect the trends in resistance across our alveolar epithelial cells. By 9 h post-pdm2009 infection (31 h; MOI 3), the resistance measurements began to decrease from the mock-infected condition and remained under 300 ohms for the duration of the experiment. Following the addition of MRSA (MOI 0.01) to the high MOI pdm2009-infected cells, resistance values across the alveolar epithelial cells began to decrease at the same time as in MRSA-alone and MOI 0.1 pdm2009-MRSA infections. However, the high MOI pdm2009-MRSA infections resulted in losses to resistance equivalent to those of the 1% triton-treated cells much more rapidly than the other infection conditions. To examine the effect of pdm2009-MRSA co-infection on distal regions of the respiratory tract, we investigated the effects of co-infection on HBEC-3KT human bronchial epithelial cells (Figure S2). The trends in loss of resistance during MRSA-alone- and pdm2009-MRSA-co-infection were largely similar to those found in A549 cells. Collectively, these data suggested that the increased damage imparted to respiratory epithelial cells by high MOI pdm2009 results in exacerbated MRSA-mediated cytotoxicity during co-infection, even at low MOIs of bacteria.

## 4. Discussion

Secondary bacterial infections can complicate both seasonal and pandemic influenza virus infections, resulting in increased morbidity and mortality [7]. During the 1918 influenza pandemic, >90% of lethal infections were complicated by bacterial co-infections and were primarily associated with *S. pneumoniae* and *S. aureus* [10]. More recently, *S. aureus*, including MRSA, has been commonly associated with influenza-bacterial co-infections, including during the 2009 pandemic, where 55% of fatal cases were associated with secondary bacterial infections [17,18,19,20]. Bacteria have the ability to sense and adapt to their surrounding environment, including during infection. This includes the modulation of replication kinetics and the synthesis of virulence factors or toxins, which can enhance both adhesive and invasive properties [48]. Here, we sought to assess the roles of host and bacterial factors directly in influenza-bacterial co-infection-mediated alveolar epithelial cell barrier dysfunction. Of note, pathophysiology associated with co-infection is thought to occur mainly in the lower respiratory tract [17]. Thus, we utilized the well-characterized alveolar epithelial adenocarcinoma A549 cells for our investigation. While it is appreciated that the potential biological implications of immortalized cell host response data must be interpreted cautiously, A549 cells provide the opportunity to assess alveolar epithelial cell responses directly in a well-characterized cell line. We have also demonstrated that the bacterial replication kinetic trends were nearly identical in the presence or absence of pdm2009 co-infection between A549 cells and HBEC-3KT cells, a normal human bronchial cell line. Further, barrier dysfunction analysis by ECIS demonstrated similar trends in loss of barrier integrity during MRSA infection alone or in conjunction with pdm2009. Future investigations will also address potential differences between non-differentiated versus differentiated/polarized A549 or primary alveolar cells.

Although we had hypothesized that pre-existing pdm2009 infection may enhance MRSA replication due to the exposure of additional bacterial binding sites on infected cells, this was not observed. Virtually identical bacterial replication kinetics were observed with no significant differences in replication at any time point. This would suggest that MRSA fitness is not altered by cellular damage or host molecule secretion (e.g., cytokines) resulting from pre-existing pdm2009 infection. This also suggests that the increased disease severity associated with influenza-bacterial co-infections is not simply due to increased bacterial burden within the lungs during co-infection.

In contrast to our replication kinetics data, the expression of MRSA virulence factors related to adhesion and invasion were selectively upregulated during the early stages of infection in pdm2009-MRSA co-infected cells as compared to bacterial infection alone. Statistical analysis demonstrated that *hla* and *spA* expression was significantly greater than all of the other virulence factors examined at 1 h post-MRSA infection; however, there were no statistically-significant differences in expression between virulence factors from 4 h onwards. Increased expression of virulence factors during pdm2009-MRSA infection as compared to MRSA-alone was inversed, as virulence factor expression in the co-infected samples was repressed as compared to MRSA-alone. This is perhaps unsurprising given that we focused on virulence factors related to bacterial adhesion and invasion. The MRSA *hla* gene codes for α-hemolysin, which forms pores in the cytoplasmic membrane of infected cells, resulting in lysis [49]. Expression of *hla* was ≥16-fold higher at 1 h post-infection, but rapidly declined by the 4-h time point. This may suggest a critical role for *hla* in the immediate stages of secondary MRSA infection pathogenesis and may contribute to co-infection pathogenesis, as α-hemolysin is related to clinical pneumonia [24,50]. Similarly, *fnbB* and *spA*, which are both involved in cell adhesion, were upregulated in the co-infected cells as compared to MRSA-alone from 1–4 h post-infection. Fibronectin-binding protein B, encoded by *fnbB*, is able to bind fibronectin, fibrinogen, and elastin in order to mediate adhesion to cells, specifically for internalization of MRSA [51,52]. Protein A, the product of *spA*, mediates binding and adhesion to airway epithelial cells, while also being able to repress innate and adaptive immune responses [53]. Our data suggest that pdm2009 infection of alveolar epithelial cells results in cellular damage and subsequent exposure of host molecules (e.g., fibrinogen, elastin, and fibronectin) in the extracellular matrix and plasma membrane, resulting in the upregulation of MRSA binding factors, including FnbB and SpA. In contrast, the relative expressions of *icaA*, *ebpS*, and *clfB* were all repressed as compared to infection with MRSA-alone throughout the course of our investigation. The *ica* locus is involved in intracellular adhesion and encodes N-acetylglucosaminyltransferase [54,55]. The *ebpS* gene is able to bind elastin in injured tissues, facilitating bacterial colonization [56], and *clfB* mediates fibrinogen [57]. Our data suggest that specific bacterial adhesion and invasion factors may provide an advantage for bacterial entry into influenza-infected cells. Future investigations of the relation between targeted inhibition of *fnbB*, *hla*, and *spA* and co-infection pathogenesis in alveolar epithelial cells are warranted and may provide important information regarding novel antimicrobial therapeutic targets. However, our data also suggest that there is likely no competitive advantage for expression of adherence and invasion-related bacterial virulence factors post-entry between pdm2009-MRSA infection and MRSA-alone infection. Further analyses of additional bacterial toxins and virulence factors may provide evidence for bacterial molecules that are related to post-bacterial adhesion/invasion co-infection pathogenesis.

Although our data suggested that the role of bacterial virulence factors in pdm2009-MRSA infection is likely important for early bacterial attachment and entry events alone, our host kinome response data suggested that host response dysregulation plays an integral role in co-infection pathogenesis. We examined this by characterizing the temporal host kinome response of alveolar epithelial cells in response to co-infection. Interestingly, the host response of co-infected cells clustered most strongly with those from pdm2009-alone infections during the early stage of infection. This was perhaps surprising, as the greatest difference in relative expression of bacterial virulence factors, including those with immunomodulatory activities (e.g., SpA), occurred early during the course of co-infection. A previous investigation by Kumar et al. demonstrated that stimulation of epithelial cells with SpA resulted in the induction of TNFα and IL8 secretion and activation of NFκB signaling [58]. While the host response in our co-infected cells was dominated by antiviral-related signaling responses during the early course of infection, GO analysis demonstrated that IL6-, IL8-, TNFα-, and NFκB-mediated signaling events were overrepresented in the co-infected samples, although absent in our MRSA-alone and pdm2009-alone infected cells. The upregulation of these signaling events corresponds with the upregulation of *spA* expression in the co-infected samples as compared to MRSA infection alone. This suggests that while the early host response during co-infection is largely dominated by the induction of antiviral responses, the upregulation of bacterial virulence factors might have an underlying influence on the induction of host cell cytotoxic responses. Interestingly, direct comparison of host kinome responses during pdm2009-MRSA infection to those from cells infected with MRSA-alone suggest that a stronger apoptotic response is found in co-infected cells as compared to bacteria-alone during the 8–12 h transition phase. This comparison supports the postulate that influenza-bacterial co-infections specifically alter host cellular responses as compared to either pathogen alone. Focused in vivo and in vitro investigations of the contributions of host response dysregulation, and in particular modulation of alveolar epithelial cell apoptosis, may provide important clues to the molecular mechanisms underlying the pathophysiology of influenza-bacterial co-infections. Future investigations will explore potential roles for the modulation of IFN-mediated cell signaling responses to pdm2009-MRSA infection pathophysiology. In particular, does modulation of the secretion of soluble host factors during pdm2009 infection impact downstream virulence factor expression patterns in MRSA?

In contrast, the kinome data (following background subtraction of the mock-infected samples) from co-infected cells clustered strongly with those derived from MRSA-alone infections from 16 h post-MRSA addition onwards, and this was reflected in strong upregulation of cell death responses (e.g., apoptosis-related pathways) in both infection conditions. From 8–12 h post-MRSA infection, host kinome data from the three different infection conditions clustered together. Taken together, our clustering data suggest that the host response transitions from an influenza- to bacterial-centric response during infection. Importantly, this transition phase in the host response corresponded with mid- to late-exponential MRSA growth. These data largely overlapped with the observations from our ECIS analysis of pdm2009-MRSA co-infection. The addition of pdm2009-alone to the alveolar epithelial cells at a low MOI of 0.1 did not result in a significant decrease in resistance, nor any negative repercussions in regards to barrier integrity and cell morphology. This reflects the majority of influenza infections in healthy adults, which do not generally result in severe disease [4,5,6]. However, the addition of high MOI pdm2009 resulted in significantly decreased barrier integrity and may reflect a relation between exacerbated disease and infectious titer of the exposure. In contrast, the addition of MRSA resulted in significant decreases in resistance and eventual loss of alveolar epithelial barrier integrity across all tested MOIs. Clinically, MRSA colonization is known to occur in healthy, young adults and may lead to overt infections, such as pneumonia [59,60]. Additionally, the development of bacterial pneumonia is known to result in inflammation of the lungs and hypoxemia, a direct result of cell barrier failure [61]. Resistance measurement trends were nearly identical between the MRSA-alone and pdm2009-MRSA infections following the addition of MRSA. These data suggest that while pdm2009, and potentially other influenza A viruses, may provide for increased adhesion and/or attachment of bacteria to the surfaces of infected epithelial cells, disruption of alveolar epithelial cell barrier function appears highly dependent on the induction of bacterial- or host cell-mediated cytotoxicity. Although the host kinome data suggest a major host-response effect, we do not exclude the possibility that bacterial-mediated cytotoxicity may still play a direct role in alveolar epithelial cell barrier dysfunction. Future investigations will focus on comparisons of host responses between these two regions of the respiratory tract during influenza-bacterial co-infection. Although our ECIS data suggest that the differential expression of bacterial virulence factors did not augment alveolar epithelial cell barrier disruption, the roles of these virulence factors in additional post-infection processes in the lung remain to be determined. These include damage to the underlying endothelium at the alveolar-capillary barrier and the disruption or attenuation of host leukocyte recruitment and immune responses within the lung.

## 5. Conclusions

Although bacterial co-infections can exacerbate influenza virus infections and result in severe or fatal disease, there is a paucity of information regarding the molecular mechanisms underlying the pathogenesis of co-infections. Our data demonstrated that while bacterial replication kinetics were similar in MRSA- and pdm2009-MRSA-infected cells, the expression of bacterial virulence factors related to adhesion and invasion were significantly upregulated during the early course of co-infection. Further, our analysis of temporal host kinome responses demonstrated that host cell signaling responses shifted from viral- to bacterial-centric throughout the course of co-infection with a transition phase in the response from 8–12 h post-MRSA addition to pdm2009 infected cells. This related well to the loss of alveolar epithelial barrier function and integrity during IAV, MRSA, or co-infection as demonstrated by ECIS.

## Figures and Tables

**Table 1:** Conservation of phosphorylation status between kinome analysis and phospho-Western blots. pdm2009-MRSA-infected A549 cell lysates 8 h post-MRSA infection were assessed by phospho-Western blot and by peptide kinome arrays.

Target	Phosphosite	Phospho-Western Blot (Fold Change)	Kinome Analysis (Fold Change)
PDGFRb	Y751	1.81	1.61
Fyn	Y420	11.12	1.54
STAT5b	Y699	9.06	2.25
Lyn	Y397	21.30	2.11
Lck	Y394	14.42	2.03
CREB	S133	2.00	2.54
β-catenin	Y654	8.97	2.39
EGFR	Y1086	2.25	2.24
Akt	S473	9.47	3.39
p38a	T180/Y182	2.65	2.13
ERK1/2	T202/Y204; T185/Y187	29.17	2.62
GSK3a/b	S21/S9	1.75	1.73
HSP60	S70	2.02	1.83
STAT3	S727	1.36	1.87
Pyk2	Y402	1.76	2.53
PLCg1	Y783	1.67	1.31
c-Jun	S63	1.87	2.49
p53	S392	3.04	2.58

## Supplementary Materials

The following are available online at http://www.mdpi.com/1999-4915/11/2/116/s1. Figure S1: MRSA replication kinetics during bacterial infection and influenza-bacterial co-infection in HBEC-3KT cells. Figure S2: pdm2009-MRSA infection decreases barrier function in bronchial epithelial cells. Table S1: MRSA RT-qPCR primer sequences; Table S2: Pathway overrepresentation analysis of host kinome responses in infected samples (8–12 h post-MRSA infection); Table S3: Pathway overrepresentation analysis of differentially-upregulated host kinome responses in pdm2009-MRSA infected cells vs. MRSA infection alone (8–12 h post-MRSA infection). Table S4: GO analysis of host kinome responses in infected samples (8–12 h post-MRSA infection).
