# Supplementary material for: Characterization of Host and Bacterial Contributions to Lung Barrier Dysfunction Following Co-infection with 2009 Pandemic Influenza and Methicillin Resistant Staphylococcus aureus
Source: Viruses. 2019 Jan 29;11(2):116. doi: 10.3390/v11020116 (PMC6409999; doi:10.3390/v11020116)
Supplement: Supplementary file 1 [file viruses-11-00116-s001.pdf]

Supplementary Data:

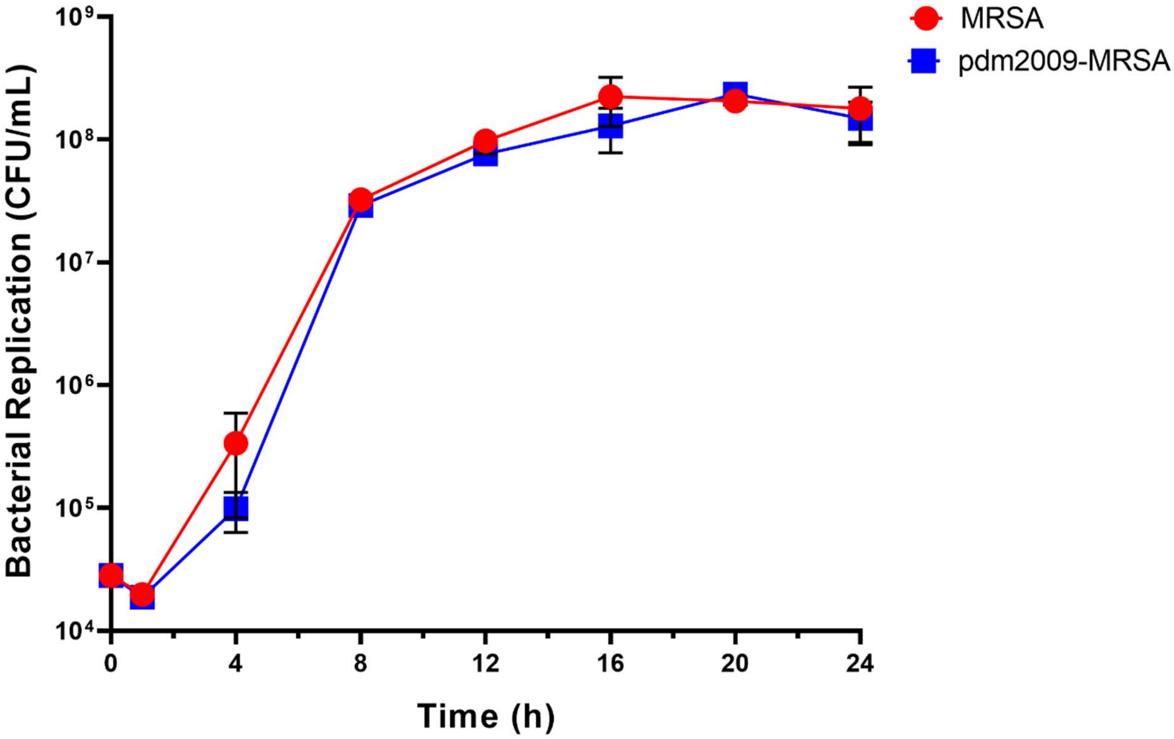

Figure S1: MRSA replication kinetics during bacterial infection and influenza-bacterial co-infection in HBEC-3KT cells. HBEC-3KT cells were infected with pdm2009 (MOI 0.1) or mock-infected followed by MRSA infection 24 hours later (MOI 0.1). HBEC-3KT cells were selectively lysed at the indicated time points and CFU were quantified by standard bacterial plating. Error bars represent SEM calculated from at least 3 biological replicates.

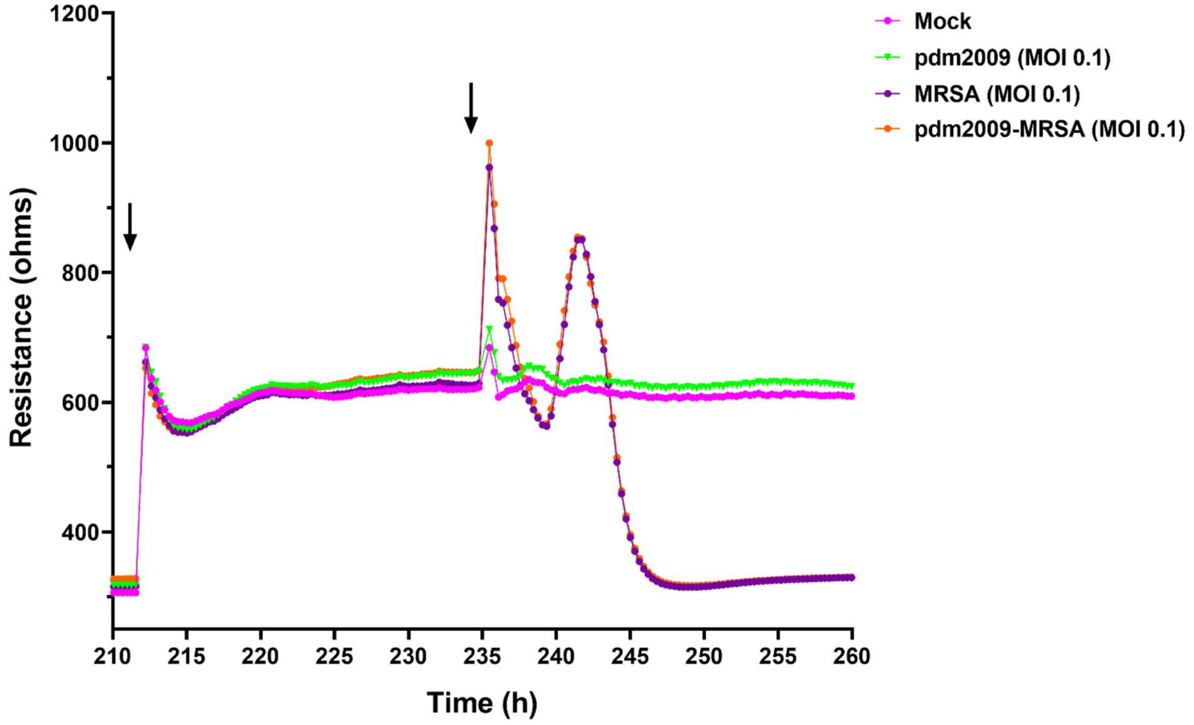

Figure S2: pdm2009-MRSA infection decreases barrier function in bronchial epithelial cells. Median resistance values have been plotted for all data points obtained during the experiment. Error bars have been removed to allow for clear visualization of all data sets but were consistent across all biological replicates. HBEC-3KT cells were plated at time 0 and grown to 90% confluency prior to initial infection with pdm2009. Cells were infected or mock-infected with pdm2009 (first arrow) and MRSA was added to cells ~24 hr later (second arrow). MRSA alone, pdm2009 alone and mock-infected time-matched conditions were also analyzed at the indicated time points. MOI values in parentheses signify the MRSA MOI utilized for infection. Resistance data represents the median of at least 3 biological replicates with at least 6 technical replicates per sample per biological replicate.

Table S1: MRSA RT-qPCR Primer Sequences

Gene	Sequence
16S	
Forward	CATGCTGATCTACGATTACT
Reverse	CCATAAAGTTGTTCTCAGTT
hla	
Forward	CTGTAGCGAAGTCTGGTGAAA
Reverse	AGATTCTTGGAACCCGGTATATG
spA	
Forward	GCTGCACCTAAGGCTAATGATA
Reverse	GATAAGAAGCAACCAGCAAACC
fnbB	
Forward	TGTCGCGCTGTATGATTGT
Reverse	GTAGAGGAAAGTGGGAGTTCAG
icA	
Forward	GCAGTAGTTCTTGTCGCATTTC
Reverse	GTTGGGTATTCCCTCTGTCTG
ebpS	
Forward	GGTGAACCTGAACCGTAGTATT
Reverse	CAGCAACAACAACGTCAAGG

Table S2: Pathway Overrepresentation Analysis of Host Kinome Responses in Infected Samples (8-12 hr Post-MRSA Infection)

A. pdm2009-MRSA Infection

Time	Signaling Pathway	Uploaded Protein Count	Pathway Upregulated P-Value
8 hr	Intrinsic Pathway for Apoptosis	12	0.039
	BH3-only proteins associate with and inactivate anti-apoptotic BCL-2 members	4	0.043
	Apoptotic signaling in response to dna damage	7	0.058
	Cell-Cell communication	7	0.058
	Amyotrophic lateral sclerosis (ALS)	10	0.061
	p75 NTR receptor-mediated signaling	10	0.061
	Alpha6Beta4Integrin	20	0.070
	Apoptosis	14	0.085
	p75(NTR)-mediated signaling	14	0.085

	Cell death signaling via NRAGE, NRIF and NADE	5	0.090
	DEx/H-box helicases activate type I IFN and inflammatory cytokines production	5	0.090
	EPH-Ephrin signaling	5	0.090
	EPHB-mediated forward signaling	5	0.090
	Metabolism of proteins	5	0.090
	Tsp-1 induced apoptosis in microvascular endothelial cell	5	0.090
	Validated targets of C-MYC transcriptional activation	5	0.090
	Viral myocarditis	5	0.090
	Apoptosis	21	0.092
	Activation of BH3-only proteins	8	0.095
	Calcium signaling by hbx of hepatitis b virus	8	0.095
	Cell surface interactions at the vascular wall	8	0.095
12 hr	Pre-NOTCH Expression and Processing	3	0.049
	Pre-NOTCH Transcription and Translation	3	0.049
	TRAF6 mediated IRF7 activation	7	0.054
	Presenilin action in Notch and Wnt signaling	8	0.078
	Basal cell carcinoma	4	0.090
	Regulation of cell cycle progression by plk3	4	0.090
	Signaling by NOTCH	4	0.090
	TRAF6 mediated IRF7 activation in TLR7/8 or 9 signaling	4	0.090
	The information processing pathway at the ifn beta enhancer	4	0.090

B. pdm2009 Infection Alone

Time	Signaling Pathway	Uploaded Protein Count	Pathway Upregulated P-Value
8 hr	IL-13 signaling	5	0.027
	Alzheimer's disease	6	0.049
	Amyotrophic lateral sclerosis (ALS)	10	0.050
	STING mediated induction of host immune responses	3	0.064
	Apoptotic signaling in response to dna damage	7	0.077

	Oxidative Stress Induced Senescence	12	0.095
12 hr	HIF-1-alpha transcription factor network	6	0.002
	Tsp-1 induced apoptosis in microvascular endothelial cell	5	0.012
	TRAF6 mediated IRF7 activation	7	0.035
	Oxidative Stress Induced Senescence	12	0.036
	Calcium signaling in the CD4+ TCR pathway	3	0.036
	Caspase cascade in apoptosis	3	0.036
	Negative regulators of RIG-I/MDA5 signaling	3	0.036
	Pertussis toxin-insensitive ccr5 signaling in macrophage	3	0.036
	Pre-NOTCH Expression and Processing	3	0.036
	Pre-NOTCH Transcription and Translation	3	0.036
	Calcineurin-regulated NFAT-dependent transcription in lymphocytes	8	0.052
	Calcium signaling by hbx of hepatitis b virus	8	0.052
	Calcium signaling pathway	8	0.052
	Endothelins	8	0.052
	Regulation of Telomerase	8	0.052
	LPA receptor mediated events	14	0.062
	Wnt signaling pathway	14	0.062
	FOXA1 transcription factor network	4	0.067
	Hypoxia-inducible factor in the cardivascular system	4	0.067
	Latent infection of Homo sapiens with Mycobacterium tuberculosis	4	0.067
	Oxidative stress induced gene expression via nrf2	4	0.067
	Phagosomal maturation (early endosomal stage)	4	0.067
	Platelet homeostasis	4	0.067
	Regulation of cell cycle progression by plk3	4	0.067
	Repression of pain sensation by the transcriptional regulator dream	4	0.067
	Signaling by NOTCH	4	0.067
	Validated transcriptional targets of AP1 family members Fra1 and Fra2	4	0.067
	Osteopontin-mediated events	9	0.072
	Regulation of nuclear SMAD2/3 signaling	9	0.072
	Colorectal cancer	22	0.089

C. MRSA Infection Alone

Time	Signaling Pathway	Uploaded Protein Count	Pathway Upregulated P-Value
8 hr	TRAF6 mediated IRF7 activation	7	0.031
	IL-13 signaling	5	0.056
	Metabolism of proteins	5	0.056
	Phagosome	5	0.056
	TRAF3-dependent IRF activation pathway	5	0.056
	Validated targets of C-MYC transcriptional activation	5	0.056
12 hr	Glucocorticoid receptor regulatory network	13	0.003
	Wnt signaling pathway	14	0.004
	Mets effect on macrophage differentiation	2	0.008
	Alzheimer's disease	6	0.010
	ErbB2/ErbB3 signaling events	12	0.014
	Oxidative Stress Induced Senescence	12	0.014
	AKT phosphorylates targets in the cytosol	7	0.017
	Apoptotic signaling in response to dna damage	7	0.017
	Cellular responses to stress	20	0.021
	Calcium signaling in the CD4+ TCR pathway	3	0.022
	Pertussis toxin-insensitive ccr5 signaling in macrophage	3	0.022
	Pre-NOTCH Expression and Processing	3	0.022
	Pre-NOTCH Transcription and Translation	3	0.022
	Unfolded Protein Response (UPR)	3	0.022
	Activation of BH3-only proteins	8	0.026
	Calcineurin-regulated NFAT-dependent transcription in lymphocytes	8	0.026
	Presenilin action in Notch and Wnt signaling	8	0.026
	Colorectal cancer	22	0.031
	Regulation of nuclear SMAD2/3 signaling	9	0.037
	BH3-only proteins associate with and inactivate anti-apoptotic BCL-2 members	4	0.042
	Basal cell carcinoma	4	0.042
	FOXA1 transcription factor network	4	0.042
	LKB1 signaling events	4	0.042
	Oxidative stress induced gene expression via nrf2	4	0.042

	Repression of pain sensation by the transcriptional regulator dream	4	0.042
	Signaling by NOTCH	4	0.042
	The information processing pathway at the ifn beta enhancer	4	0.042
	Amyotrophic lateral sclerosis (ALS)	10	0.049
	Direct p53 effectors	10	0.049
	Cellular Senescence	17	0.051
	T cell receptor signaling pathway	18	0.062
	AP-1 transcription factor network	11	0.064
	Phosphoinositides and their downstream targets	11	0.064
	Role of Calcineurin-dependent NFAT signaling in lymphocytes	11	0.064
	Signaling mediated by p38-alpha and p38-beta	11	0.064
	Activation of BAD and translocation to mitochondria	5	0.066
	Atm signaling pathway	5	0.066
	IL12 signaling mediated by STAT4	5	0.066
	Metabolism of proteins	5	0.066
	Regulation of nuclear beta catenin signaling and target gene transcription	5	0.066
	S1P2 pathway	5	0.066
	Tsp-1 induced apoptosis in microvascular endothelial cell	5	0.066
	ATF-2 transcription factor network	12	0.081
	Downstream signaling in naïve CD8+ T cells	12	0.081
	Intrinsic Pathway for Apoptosis	12	0.081
	Activation of the AP-1 family of transcription factors	6	0.093
	Aurora A signaling	6	0.093
	Cadmium induces dna synthesis and proliferation in macrophages	6	0.093
	FOXM1 transcription factor network	6	0.093
	HIF-1-alpha transcription factor network	6	0.093

Table S3: Pathway Overrepresentation Analysis of Differentially Upregulated Host Kinome Responses in pdm2009-MRSA infected cells vs. MRSA infection alone (8-12 hr Post-MRSA Infection)

Time	Signaling Pathway	Uploaded protein count	Pathway up-regulated p-value
8hr	Cell death signalling via NRAGE, NRIF and NADE	5	0.005
	Caspase Cascade in Apoptosis	10	0.007
	p75 NTR receptor-mediated signalling	10	0.007
	Intrinsic Pathway for Apoptosis	12	0.014
	Caspase cascade in apoptosis	3	0.022
	Apoptosis	14	0.026
	p75(NTR)-mediated signaling	14	0.026
	CDC42 signaling events	15	0.033
	Toxoplasmosis	31	0.037
	BH3-only proteins associate with and inactivate anti-apoptotic BCL-2 members	4	0.042
	Stress induction of hsp regulation	4	0.042
	TRAF6 mediated IRF7 activation in TLR7/8 or 9 signaling	4	0.042
	p75NTR recruits signalling complexes	4	0.042
12 hr	Cell death signalling via NRAGE, NRIF and NADE	5	0.003
	p75 NTR receptor-mediated signalling	10	0.016
	TRAF6 mediated IRF7 activation in TLR7/8 or 9 signaling	4	0.017
	TRAF6 mediated IRF7 activation	7	0.018
	Phagosome	5	0.038

Table S4: GO Analysis of Host Kinome Responses in Infected Samples (8-12 hr Post-MRSA Infection)

A. pdm2009-MRSA Infection

Time	Signaling Pathway	Uploaded Protein Count	Pathway Upregulated P-Value
8 hr	regulation of inflammatory response	6	0.003
	positive regulation of release of cytochrome c from mitochondria	5	0.011

	positive regulation of interleukin-6 production	3	0.013
	intrinsic apoptotic signaling pathway	12	0.037
	intrinsic apoptotic signaling pathway in response to DNA damage	7	0.055
	cellular protein metabolic process	5	0.087
	negative regulation of protein catabolic process	5	0.087
	neuron apoptotic process	5	0.087
	positive regulation of interferon-alpha production	5	0.087
	positive regulation of peptidyl-tyrosine phosphorylation	5	0.087
	response to progesterone	5	0.087
	positive regulation of cell migration	11	0.087
	extrinsic apoptotic signaling pathway in absence of ligand	8	0.091
	release of cytochrome c from mitochondria	8	0.091
12 hr	positive regulation of neuron apoptotic process	10	0.004
	cellular response to DNA damage stimulus	13	0.017
	positive regulation of interferon-alpha production	5	0.018
	positive regulation of tumor necrosis factor production	6	0.033
	transforming growth factor beta receptor signaling pathway	11	0.043
	ATP catabolic process	3	0.048
	ER overload response	3	0.048
	ion transport	3	0.048
	positive regulation of interleukin-6 production	3	0.048
	positive regulation of interleukin-8 production	3	0.048
	positive regulation of type I interferon-mediated signaling pathway	3	0.048
	protein localization to nucleus	3	0.048

	proteolysis	3	0.048
	response to unfolded protein	3	0.048
	B cell homeostasis	7	0.052
	intrinsic apoptotic signaling pathway in response to DNA damage	7	0.052
	regulation of apoptotic process	12	0.059
	release of cytochrome c from mitochondria	8	0.076
	positive regulation of apoptotic process	24	0.076
	transcription from RNA polymerase II promoter	13	0.077
	B cell activation	4	0.088
	T cell homeostasis	4	0.088
	erythrocyte differentiation	4	0.088
	mammary gland development	4	0.088
	negative regulation of protein phosphorylation	4	0.088
	positive regulation of interferon-beta production	4	0.088
	regulation of protein phosphorylation	4	0.088
	response to UV	4	0.088
	transport	4	0.088
	type I interferon biosynthetic process	4	0.088

B. pdm2009 Infection Alone

Time	Signaling Pathway	Uploaded Protein Count	Pathway Upregulated P-Value
8 hr	neuron apoptotic process	5	0.030
	regulation of transcription from RNA polymerase II promoter	13	0.037
	defense response to Gram-positive bacterium	3	0.067
	multicellular organism growth	3	0.067

	positive regulation of interleukin-6 production	3	0.067
	positive regulation of protein oligomerization	3	0.067
	regulation of mitochondrial membrane permeability involved in apoptotic process	3	0.067
	in utero embryonic development	15	0.068
	inflammatory response	20	0.073
12 hr	positive regulation of tumor necrosis factor production	6	0.021
	response to cAMP	6	0.021
	transforming growth factor beta receptor signaling pathway	11	0.025
	cellular response to calcium ion	3	0.035
	defense response to Gram-positive bacterium	3	0.035
	negative regulation of type I interferon production	3	0.035
	nitric oxide biosynthetic process	3	0.035
	receptor-mediated endocytosis	3	0.035
	response to immobilization stress	3	0.035
	somitogenesis	3	0.035
	defense response to virus	8	0.050
	release of cytochrome c from mitochondria	8	0.050
	defense response to bacterium	4	0.066
	interaction with host	4	0.066
	phagosome maturation	4	0.066
	positive regulation of interferon-beta production	4	0.066
	positive regulation of vasodilation	4	0.066
	response to UV	4	0.066
	transport	4	0.066

	regulation of cell proliferation	15	0.075
	negative regulation of cell proliferation	22	0.085
	positive regulation of neuron apoptotic process	10	0.093
	response to hypoxia	10	0.093

C. MRSA Infection Alone

Time	Signaling Pathway	Uploaded Protein Count	Pathway Upregulated P-Value
8 hr	positive regulation of interferon-alpha production	5	0.006
	defense response to Gram-positive bacterium	3	0.007
	negative regulation of protein phosphorylation	4	0.025
	positive regulation of interferon-beta production	4	0.025
	regulation of immune response	4	0.025
	cellular protein metabolic process	5	0.054
	neuron apoptotic process	5	0.054
	oxidation-reduction process	5	0.054
	positive regulation of peptidyl-tyrosine phosphorylation	5	0.054
	positive regulation of neuron projection development	6	0.093
	protein homooligomerization	6	0.093
	regulation of inflammatory response	6	0.093
12 hr	ER overload response	3	0.001
	positive regulation of mitochondrial outer membrane permeabilization involved in apoptotic signaling pathway	4	0.002
	transcription from RNA polymerase II promoter	13	0.002
	Wnt signaling pathway	8	0.002

	release of cytochrome c from mitochondria	8	0.002
	negative regulation of canonical Wnt signaling pathway	5	0.005
	positive regulation of release of cytochrome c from mitochondria	5	0.005
	positive regulation of transcription from RNA polymerase II promoter	39	0.007
	positive regulation of apoptotic process	24	0.009
	positive regulation of transcription, DNA-templated	24	0.009
	activation of signaling protein activity involved in unfolded protein response	3	0.021
	cellular response to calcium ion	3	0.021
	endoplasmic reticulum unfolded protein response	3	0.021
	negative regulation of glycogen biosynthetic process	3	0.021
	positive regulation of protein catabolic process	3	0.021
	positive regulation of protein oligomerization	3	0.021
	regulation of mitochondrial membrane permeability	3	0.021
	regulation of mitochondrial membrane permeability involved in apoptotic process	3	0.021
	regulation of transcription, DNA-templated	37	0.023
	extrinsic apoptotic signaling pathway in absence of ligand	8	0.025
	positive regulation of intrinsic apoptotic signaling pathway	9	0.035
	positive regulation of protein insertion into mitochondrial membrane involved in apoptotic signaling pathway	9	0.035
	epithelial to mesenchymal transition	4	0.041
	glycogen metabolic process	4	0.041
	positive regulation of neuron apoptotic process	10	0.048
	sequence-specific DNA binding	17	0.049
	transforming growth factor beta receptor signaling pathway	11	0.062
	cellular protein metabolic process	5	0.064
	cellular response to UV	5	0.064
	neuron apoptotic process	5	0.064

	response to progesterone	5	0.064
	intrinsic apoptotic signaling pathway	12	0.078
	regulation of sequence-specific DNA binding transcription factor activity	6	0.091
	response to cAMP	6	0.091
	cellular response to DNA damage stimulus	13	0.096
	negative regulation of transcription, DNA-templated	13	0.096
